# *Tiarajudens eccentricus* and *Anomocephalus africanus*, two bizarre anomodonts (Synapsida, Therapsida) with dental occlusion from the Permian of Gondwana

**DOI:** 10.1098/rsos.150090

**Published:** 2015-07-15

**Authors:** Juan Carlos Cisneros, Fernando Abdala, Tea Jashashvili, Ana de Oliveira Bueno, Paula Dentzien-Dias

**Affiliations:** 1 Centro de Ciências da Natureza, Universidade Federal do Piauí, Teresina, Brazil; 2 Evolutionary Studies Institute, University of the Witwatersrand, Johannesburg, South Africa; 3 Departamento de Paleontologia e Estratigrafia, Universidade Federal do Rio Grande do Sul, Porto Alegre, Brazil; 4 Laboratório de Paleontologia e Paleoceanografia, Universidade Federal do Rio Grande, Rio Grande, Brazil

**Keywords:** Therapsida, Anomodontia, herbivory, dental occlusion, agonistic behaviour, Permian

## Abstract

Anomodontia was a highly successful tetrapod clade during the Permian and the Triassic. New morphological information regarding two bizarre basal anomodonts is provided and their palaeoecological significance is explored. The osteology of the recently discovered *Tiarajudens eccentricus* Cisneros *et al*. 2011, from the Brazilian Permian, is described in detail. The taxon exhibits unusual postcranial features, including the presence of gastralia. Additional preparation and computed tomography scans of the holotype of *Anomocephalus africanus* Modesto *et al*. 1999 discovered in the Karoo Basin of South Africa allow a reappraisal of this genus. *Anomocephalus* is similar to *Tiarajudens* with regard to several traits, including a battery of large, transversally expanded, palatal teeth. Molariform teeth are present in the mandible of the African taxon, providing additional insight into the function of the earliest tooth-occlusion mechanism known in therapsids. At least two waves of tooth replacement can be recognized in the palate of *Anomocephalus*. The outsized, blade-like caniniforms of the herbivorous *Tiarajudens* allow several non-exclusive ecological interpretations, among which we favour intraspecific display or combat. This behaviour was an alternative to the head-butting practised by the contemporary dinocephalians. Combat specializations that are considered typical of Cenozoic herbivores likely evolved during the Middle Permian, at the time the first communities with diverse, abundant tetrapod herbivores were being assembled.

## 1. Introduction

Most of our knowledge on basal therapsids comes from two main areas, the South African Karoo Basin and the Russian Platform. A better record of early members of this group is important to understand the faunal and ecological changes that took place in the Middle Permian, namely the turnover of basal synapsid-dominated faunas to therapsid-dominated faunas [1–3] and the establishment of modern terrestrial vertebrate ecosystems [4–6]. The exploration of new geographical areas and the discovery of new basal synapsids emerge as a necessity to improve our record of early members of this group [7], to resolve higher-level relationships among major therapsid clades and also to provide clues as to where therapsids actually originated.

**Figure 1.**
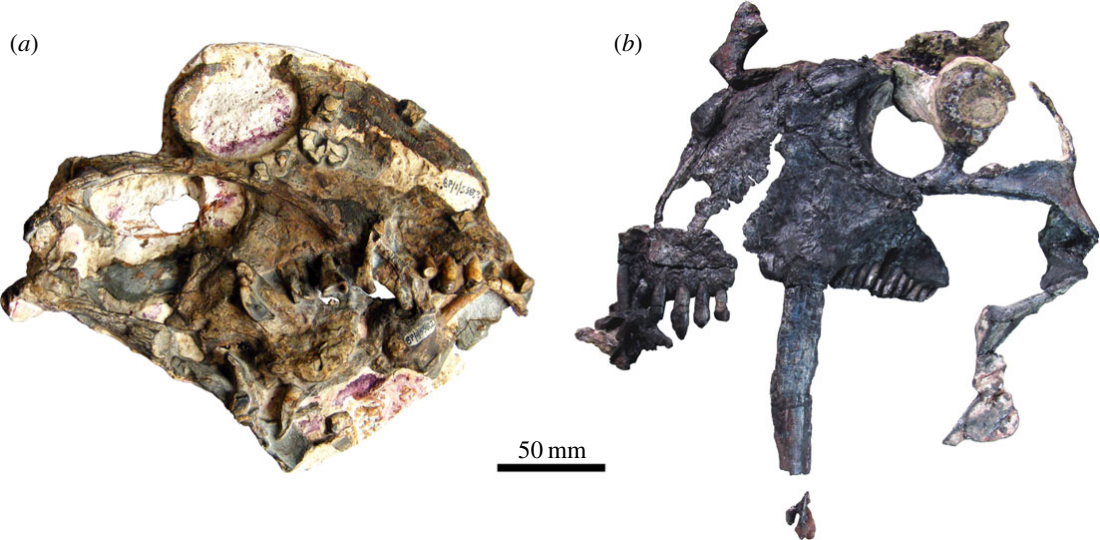
(*a*) *Anomocephalus africanus* (BP-1-5582) from the Middle Permian of South Africa, cranium, right lateral view. (*b*) *Tiarajudens eccentricus* (UFRGS PV393P), from the Middle Permian of Brazil, cranium, left lateral view.

As a result of fieldwork in the Permian of Brazil, a very unusual herbivorous therapsid was found in Guadalupian rocks of the Paraná Basin, in the state of Rio Grande do Sul, in 2009. The new species, *Tiarajudens eccentricus* [8] (figure 1*b*), was revealed as not only the earliest therapsid capable of dental occlusion, but also combining a suit of characters highly unusual for a herbivore, such as molariform teeth in the palate and huge sabre-caniniforms.

The discovery of *T. eccentricus* sheds new light on another peculiar therapsid, the basal anomodont *Anomocephalus africanus* (figure 1*a*), recovered more than 10 years earlier in the Karoo Basin [9]. This taxon was originally considered to be the most basal anomodont, but several important features of its anatomy were overlooked, probably due to its poor preservation and incomplete preparation. Our new interpretation of the specimen shows that it shares several key characteristics with *Tiarajudens*, including molariform teeth in the palate. Here, we provide an anatomical account of the cranium and the postcranium of *T. eccentricus*, we re-evaluate its sister taxon *A. africanus*, and explore the ecological significance of these two species.

## 2. Material and methods

**Figure 2.**
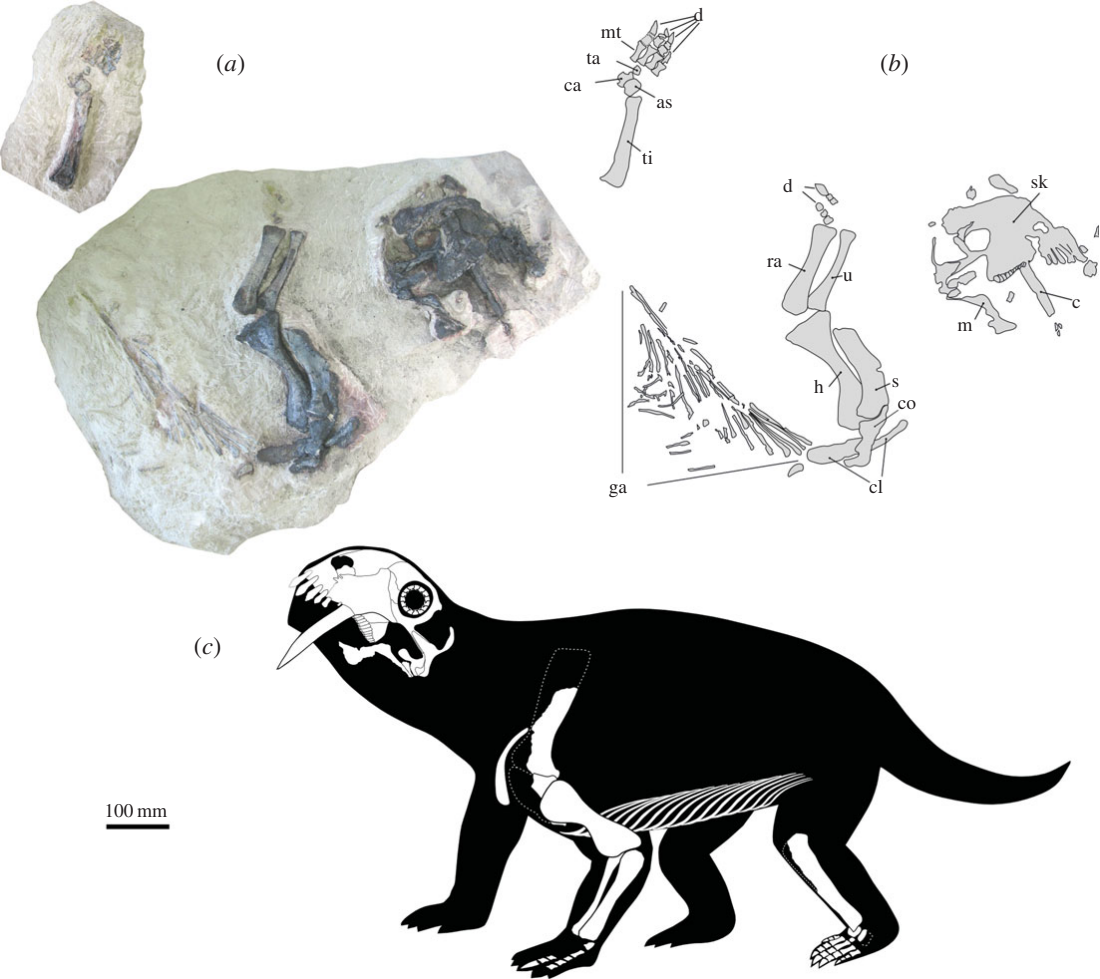
Skeleton of *T. eccentricus*. (*a*) Sandstone blocks containing articulated skeletal material. (*b*) Schematic drawing showing the identity of the preserved elements. (*c*) Skeletal reconstruction. as, astragalus; c, caniniform; ca, calcaneum; cl, clavicle; co, coracoid; d, digits; ga, gastralia; h, humerus; m, mandible; mt, metatarsals; ra, radius; sk, skull; ti, tibia; ta, tarsal.

The specimen of *T. eccentricus* (figure 2) was found partially articulated in a sandstone lens. Half the skull was lying on its left lateral surface, exposed in medial view and the left, partial lower jaw was found articulated with the skull. The cranium was found less than 200 mm from a partial left pectoral girdle. Associated with the girdle was the left limb, including some bones from the manus. An isolated left tibia with the pes was also recovered. The foot elements are contained in two slabs of rock that were accidentally discovered and opened up during retrieval of the specimen. They were found a few centimetres from the gastralia. No other skeletal elements were recognized. The whole specimen underwent flattening during preservation.

Additional preparation was carried out on the cranium of *A. africanus* in order to clarify details of its dentition. Due to the fact that the medial surface of this partial skull was embedded in plaster, a window was opened in order to allow cleaning of the medial areas of the tooth-bearing bones of the snout, palate and lower jaw. Further preparation was also done on the lateral surface of some teeth.

The description of the humerus is considering the major axis of the bone parallel to the body, the head located in the proximal margin and the epicondyles in the distal margin. The radius–ulna axis is interpreted as perpendicular to that of the humerus, the articulation surface of the ulna with the humerus being directed anterodorsally.

An X-ray computed tomography (CT) scan was performed in order to explore details of the internal anatomy of the two species (see electronic supplementary material). The skull and block containing foot bone elements of *T. eccentricus* and the skull of *A. africanus* were CT-scanned by Dr Sulman and Partners, Rosebank Clinic, Johannesburg, using a Toshiba-MEC CT3 scanner. All specimens were scanned together using 0.5 mm slice and Open SKULL 0.5 protocol under 120 mAs energy, 1000 exposure time and 400 X-ray tube current. The raw data were reconstructed using ‘Bone’ kernel. The Dicom images were registered on 16 bit per pixel and 512 based matrix. Reconstructed images correspond to 0.53×0.53 mm. Each skull and its postcranial elements were segmented separately with the help of Avizo^®^ 7.0 software (Visualization Sciences Group, Mérignac cedex, France VSG, SAS).

Both slabs containing foot elements of *Tiarajudens* were scanned, except for the first ray. One of the blocks including the metatarsals and phalanges was sectioned longitudinally on a horizontal plane during the collection of the specimen. In order to reconstruct the elements contained in the two slabs, first we segmented out dense bone material from both slabs, using an 1800 UH threshold. For each portion of the sectioned elements, we defined matching planes. With the help of Avizo^®^ 7.0 software, we performed rotation and translation to fit the planes together, and this produced the best fitted reconstruction for each bone. The final volume renderings for illustrations of the reconstructed metatarsals and phalanges were produced with the help of the Volume Graphics software VG Studio Max v. 2.2.

### 2.1 Institutional abbreviations

UFRGS, Universidade Federal do Rio Grande do Sul, Porto Alegre, Brazil. BP, Evolutionary Studies Institute (formerly Bernard Price Institute for Palaeontological Research), University of the Witwatersrand, Johannesburg, South Africa. PIN, Palaeontological Institute, Russian Academy of Sciences, Moscow, Russia. SAM, Iziko South African Museum, Cape Town, South Africa. NMQR, National Museum, Bloemfontein, South Africa.

## 3. Systematic palaeontology

Therapsida Broom, 1905 [10].

Anomodontia Owen, 1859 [11].

Anomocephaloidea Cisneros *et al*., 2011 [8].

*Anomocephalus africanus* Modesto, Rubidge and Welman, 1999 [9].

### 3.1 Holotype

BP-1-5582, partial cranium and unprepared postcranium (figure 1*a*; see electronic supplementary material, video S1).

### 3.2 Locality

Vleikraal Farm, near Williston, Northern Cape, South Africa.

### 3.3 Horizon

The sandstone horizon, which produced the fossil, is likely referable as the informal Kopjesfontein Member [12,13] of the Abrahamskraal Formation, Beaufort Group. Lower part of the *Tapinocephalus* AZ, Middle Permian (Guadalupian).

### 3.4 Emended diagnosis

Large basal anomodont distinguished from other anomodonts by the presence of a tall, tongue-shaped coronoid eminence. It differs further from most other anomodonts (with the exception of *T. eccentricus*) by the presence of a series of transversally expanded teeth (the average ratio of labiolingual–mesiodistal length=3:1) in the ectopterygoid and/or pterygoid. It is distinguished from *T. eccentricus* by the absence of maxillary caniniforms.

*Tiarajudens eccentricus* Cisneros, Abdala, Rubidge, Dias and Bueno, 2011 [8].

### 3.5 Holotype

UFRGS PV393P, partially articulated cranium and postcranium (figures 1*b* and 2; see electronic supplementary material, video S2).

### 3.6 Locality

**Figure 3.**
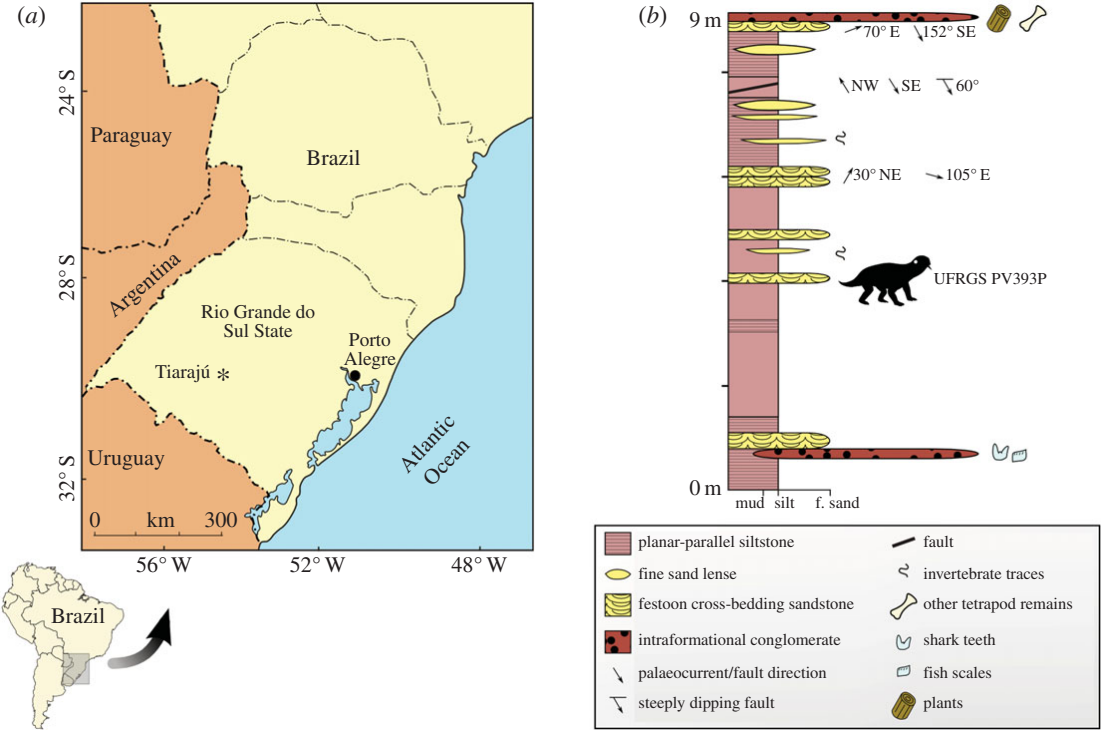
Provenance of *T. eccentricus*. (*a*) Location of the Tiarajú District in Rio Grande do Sul State, southern Brazil. (*b*) Sedimentological log of the type locality.

Tiarajú District, São Gabriel Municipality, Rio Grande do Sul State, Brazil (figure 3*a*).

### 3.7 Horizon

The exposure is representative of the Morro Pelado Member of the Rio do Rasto Formation, Middle Permian (Guadalupian) [14,15]. The outcrop is a 9 m thick succession of massive, sometimes planar-parallel siltstone, intercalated by sandstone lenses in the upper part (figure 3*b*). There are two layers of intraformational conglomerate. At the base of the section, the first conglomerate is formed by granule-sized grains, bearing xenacanthid teeth and abundant, actinopterygian scales, whereas at the top, the conglomerate is composed of pebble-sized clasts, and produces fossil wood and tetrapod bones. Sandstone layers bearing cross-festonate stratification occur along the exposure. The sedimentary sequence can be classified as a floodplain, cut by small fluvial channels.

### 3.8 Diagnosis

Large basal anomodont distinguished by the presence of extremely large maxillary caniniforms, comprising more than 120% of the maximum snout (antorbital) height and more than 60% of the total skull length, reniform in proximal basal cross section, and featuring enamel. It differs from other anomodonts, except *A*. *africanus*, by having a row of transversally expanded palatal teeth (average ratio of labiolingual–mesiodistal length=3:1), located in the ectopterygoid and pterygoid, showing uneven wear facets and long roots [8].

## 4. Description of *Tiarajudens eccentricus*

### 4.1 General remarks

**Figure 4.**
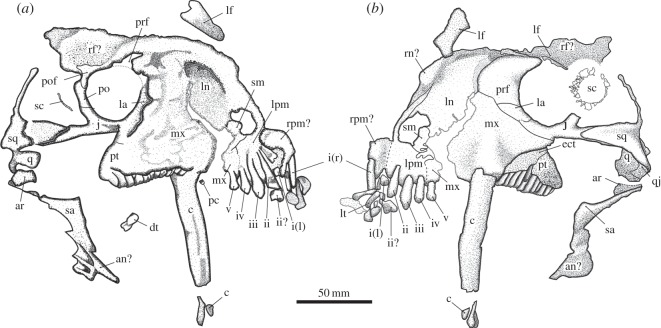
Cranium of *T. eccentricus*, technical drawings. (*a*) Left medial view. (*b*) Left lateral view. i–iv, tooth positions; an, angular; ar, articular; c, caniniform; dt, disarticulated teeth; ect, ectopterygoid; j, jugal; l, left; la, lacrimal; lf, left frontal; ln, left nasal; lpm, left premaxilar; lt, lower jaw tooth; mx, maxilla; pc, precaniniform; po, postorbital; pof, postfrontal; prf, prefrontal; pt, pterygoid; q, quadrate; qj, quadratojugal; r, right; rf, right frontal; rn, right nasal; rpm, right premaxilar; sa, surangular; sc, scleral ossicles; sm, septomaxilla; sq, squamosal.

**Figure 5.**
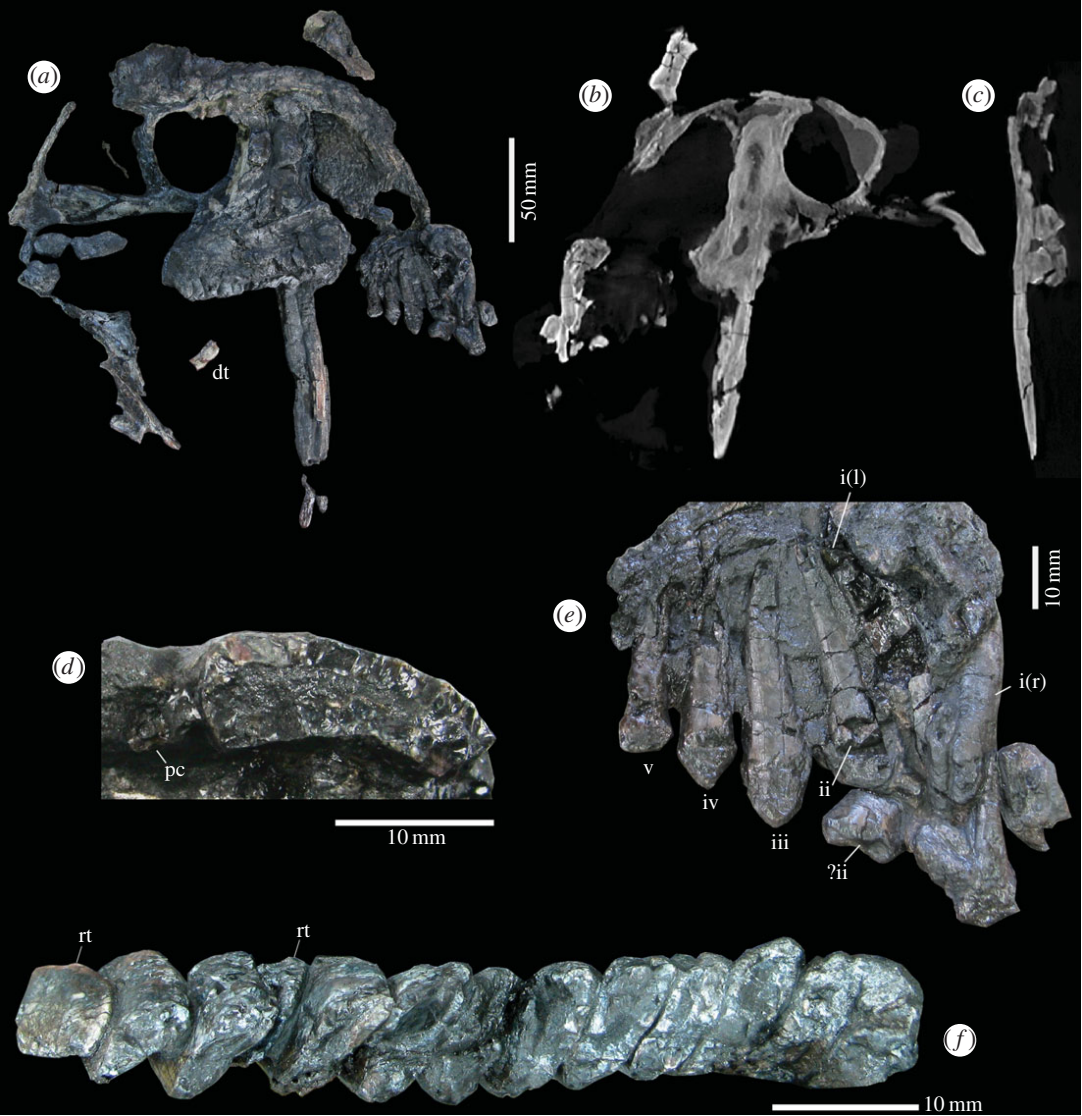
Cranium and dentition of *T. eccentricus*. (*a*) Cranium, left lateral view. (*b*) Cranium, parasagittal section (CT scan), right view. (*c*) Cranium, transversal section (CT scan) at the level of the caniniform, posterior view. (*d*) Ventral view of the base of the left caniniform (exposed due to a fracture) and a precaniniform, anterior to the left. (*e*) Incisiform teeth, medial view. (*f*) Left palatal teeth, occlusal view, anterior to the right. i–iv, tooth positions; dt, disarticulated teeth; l, left; pc, precaniniform; r, right; rt, replacement tooth.

The skull of *Tiarajudens* (figures 1*b*, 4 and 5) is relatively large (approx. 225 mm long) for a basal anomodont. The preorbital length is only slightly shorter than the postorbital skull length (approx. 45% of cranial length). As typical in anomodonts, the maximum skull height (107 mm) is near the anterior margin of the orbit, and shallows posterior to the orbit, giving the skull a domed profile in lateral view. The orbit is relatively large, being slightly longer anteroposteriorly than the temporal fenestra (approx. 51 mm×approx. 47 mm, respectively). The surface of the bones is well preserved, being smooth and lacking any trace of ornamentation.

### 4.2 Antorbital region

The left premaxilla (figures 4 and 5) has a deep dentigerous, horizontal ramus, bearing at least four teeth (see below), this portion forming the ventral margin of the external naris. There is a small anterior premaxillary foramen at the base of the ascending process. The premaxilla features a slender ascending process that forms most of the anterior margin of the external naris, its posterior half being confined by the nasal. The premaxilla–maxilla suture is partially visible, located on the level of the posterior margin of the external naris. There is a small portion of the right premaxilla, lying on the same plane of its left counterpart, and this includes at least one right incisor. The external naris is reniform, its major axis oriented anteroventrally to posterodorsally. Its posterior border is formed by the small septomaxilla, and the dorsal margin by the nasal. The nasal is wide and has a smooth surface. The most anterior portion of the nasal is an anterior spur which is in contact with the ascendant process of the premaxilla, expanding posteriorly. Dorsal to the spur, the nasal widens considerably. Most of its contact with the maxilla is represented by a fracture in the snout. This crack occurred along the suture of the two bones. Its contact with the frontal appears to occur anterior to the orbital rim. The septomaxilla has an elongated, uniform facial exposure ending before the dorsal margin of the external naris. Its dorsal portion gives rise to a short, bulbous, anteromedial projection, constricted ventrally by a shallow lateromedial sulcus (figure 4*a*). The anterior margin of the maxilla is preserved as a free margin due to a fracture. Only a small anteroventral portion of this bone is joined to the posterior border of the premaxilla. The maxilla has a wide, mostly flat, exposed lateral surface which features a triangular swelling at the base of the caniniform. The dorsal margin of the maxilla is extended to a level which is at midheight of the orbit. Dorsoposteriorly, the maxilla contacts the lacrimal and the prefrontal and extends posteriorly to the midlength of the orbit. The ventral margin of the maxilla rises as a prominent ridge, posterior to the caniniform, forming the anterior root of the zygoma.

Since the description of *Tiarajudens* [8], the relationships between the prefrontal, lacrimal and jugal (figure 4*b*) have been re-evaluated. Most of the dorsal portion of the prefrontal appears to be covered by an unidentified bone. The prefrontal seems to be a mostly flat, roughly triangular bone, condition which is probably the result of compression. Its anterodorsal border is strongly convex. The anterior half of this margin represents the suture with the nasal, whereas the remaining part of this border is likely the contact with the area left by the missing anterior portion of the frontal. The anteroventral margin of the prefrontal can be subdivided in two short, concave borders, the most anterior contacting the maxilla, and the posterior contacting the lacrimal and reaching the orbital rim. The posterior margin of the prefrontal forms half of the anterior portion of the orbital rim. The lacrimal is diamond shaped. Its longer contact is with the maxilla, along its anteroventral margin. Dorsally, it is limited by the prefrontal, posteriorly it forms a small portion of the orbital rim, and ventrally it shares a short suture with the jugal. The frontal is poorly preserved and appears to be widely exposed, forming the dorsal margin of the orbit. What is probably a portion of the right frontal was found displaced, dorsal to the nasal.

### 4.3 Temporal region

The anterior portion of the suborbital zygoma (figure 4), formed by the maxilla and jugal, is higher than the posterior portion, formed only by the jugal. The latter has an anterior extension that surpasses the orbital rim, ending at the level of the caniniform root. The jugal forms the basal portion of the postorbital bar, being of comparable thickness to the zygomatic arch immediately posterior to this bar. The postorbital bar is slightly recurved and of uniform width through all its extension. The contact between the jugal and the postorbital is hidden laterally by the displaced sclerotic ring, and medially it is not visible due to weathering. The temporal portion of the zygoma is formed by a small contribution of the jugal, immediately behind the postorbital bar and by the large anterior projection of the squamosal that laterally overlaps the former. The zygomatic arch in the temporal region starts as a cylindrical bone that expands posteriorly as a lamina. The ventral margin of the bar is straight, except along its most posterior portion which expands ventrally, whereas its dorsal margin gradually rises backwards. The zygomatic process of the squamosal is triangular and has a shallow longitudinal sulcus parallel to its dorsal margin. The squamosal does not show the lateral flange for the attachment of lateral external adductor muscle as in dicynodonts, presenting a thin projection that forms the posterior margin of the temporal opening (figure 4). The squamosal produces a ventral, anteriorly concave recess, for the articulation with the quadrate and the quadratojugal. The quadrate and the quadratojugal are preserved *in situ*. Their location in the skull is at the posterior end of the zygoma. The quadrate is partially exposed in lateral view as an irregular lamella. Its dorsoventral dimension is roughly equivalent to the preserved anteroposterior length, even though the anterior border is damaged. Its posterior portion is covered by the ventral process of the squamosal and the quadratojugal. A robust medial quadrate condyle is visible in medial view, where it appears as a subrectangular structure, being slightly longer than tall. The small quadratojugal overlies the quadrate in lateral aspect. It appears as an oblique rectangle with a concave anterodorsal margin. Its posterodorsal end lies below the end of the squamosal ventral process.

### 4.4 Palate

Below the orbit, there is a tooth-bearing triangular structure (figures 4 and 5*a*), oriented posteromedially in relation to the lateral margin of the cranium, formed by the ectopterygoid and the pterygoid. The first element is a strip of bone carrying four teeth that are visible laterally and two more anterior teeth, hidden by the caniniform, which are visible in palatal view (figure 5*f*). Only the most anterior lateral portion of the pterygoid is preserved, as a triangular structure. It bears seven teeth, the three most posterior being inset from the lateral margin of the bone by a small platform.

### 4.5 Sclerotic ring

The articulated ring of sclerotic ossicles (figure 4*b*) is displaced over the postorbital bar. It consists of probably 19 delicate elements. These are quadrangular bones. In some of them, a short acute process is visible adjacent to the inner margin, overlapping the neighbouring element.

### 4.6 Mandible

Most of the lower jaw (figures 4 and 5*b*) is lost, only three bones being partially preserved. A portion of the dorsal border of the articular is visible. The dorsal margin of the surangular, preserved as a long bar with an expanded anterior end, is in contact ventrally with a plate that is here interpreted as a part of the reflected lamina.

### 4.7 Dentition

*Tiarajudens eccentricus* has at least five prominent, leaf-shaped, upper incisiform teeth (figures 4 and 5*a*,*e*), located in the premaxilla and maxilla. The bone fracture that separated the snout is located immediately behind the fifth incisiform, and the possibility remains that at least one other small tooth may have been present. All incisiforms possess conical, closed roots, are exposed in lingual view (figure 5*e*) [8], fig. 1*f* and feature a thin layer of enamel. A poorly developed heel is present, on the medial surface, at the base of well-preserved crowns. The first incisiform present appears to be the largest tooth, and it belongs to the right premaxilla. The root and part of the neck are exposed labially, whereas the crown is covered by a displaced tooth that probably belongs to the lower jaw. Its lanceolate crown, however, is visible lingually, featuring a weak, inverted V-shaped, heel (figure 5*e*). The first left incisiform is fragmented, its length and shape being comparable to the next posterior element. The second upper incisiform is well preserved except for the missing crown. Below it, the crown of a flat, transversally expanded tooth, probably from the lower jaw (figure 5*e*), is preserved. The third incisiform is somewhat wider, and probably longer than the second. It is lanceolate, possessing a labially convex crown, with its largest width on its base. An undulation along the edges is visible on the distal border of its crown. The fourth tooth is notably smaller than the previous one, being also lanceolate (unfortunately the tip was damaged during preparation, but it is recorded in figure 5*e* as originally found). Its crown possesses an inverted V-shaped, faint ridge on its medial surface that runs parallel to the apical margin. The fifth tooth, located in the maxilla, is smaller than the fourth. It features a labially convex, longitudinally faceted, crown. Contrary to other incisiforms, it lacks an acute apex, probably as a result of wear, its maximum width being recorded close to its apical border. The subcircular alveolus and the partially exposed root of a very small precaniniform (approx. 2 mm in cross section), located some 2 mm anterior to the caniniform, is visible in lingual view (figure 5*d*). Considering the possibility of a missing incisiform in the area of the bone fracture that separated the snout, a total count of seven upper teeth before the caniniform seems likely.

There is an extremely large, nearly straight caniniform (approx. 120 mm) located just anterior to the level of the orbital rim. It is rather thin (figure 5*c*), measuring *ca* 7 mm maximum thickness at the base. It possesses also a deep root (approx. 85 mm) that extends up in front of the orbit, close to the roof of the skull (figure 5*b*). It is not possible to assess with confidence if this root is open or closed. The caniniform is reniform in basal cross section (mesiodistal length: 18 mm, figure 5*d*), exhibiting a well-developed longitudinal sulcus that runs through most of its lingual surface (figure 5*a*). The labial surface of the caniniform is slightly mesiodistally convex, featuring longitudinal facets and widespread, thin enamel. Proximally, its mesial border is a flat area, which progressively thins distally, forming a sharp ridge. No serrations were found along its margins.

**Figure 6.**
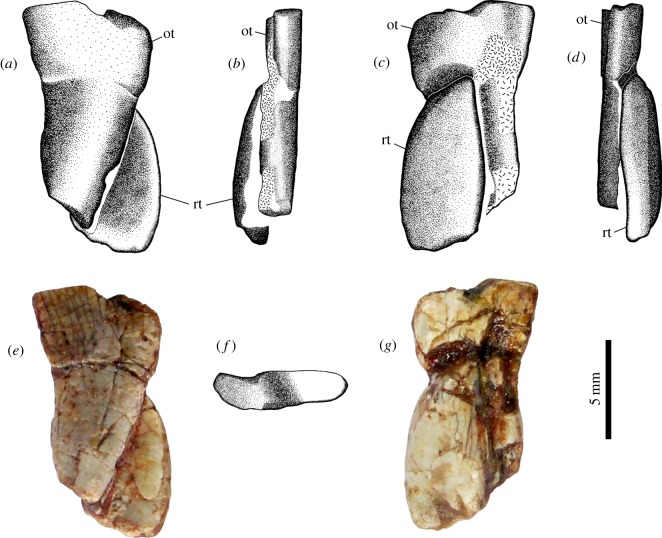
Isolated molariforms of *T. eccentricus*, probably right palatal teeth, including a replacement element. (*a*,*e*) ?Distal view. (*b*) ?Labial view. (*c*,*g*) ?Medial view. (*d*) ?Lingual view. (*f*) Old tooth, occlusal view. ot, old tooth; rt, replacement tooth.

A straight row of 13 large, transversally expanded, palatal teeth (figure 5*f*) are present. The axes of the crowns are oblique in relation to the skull midline, oriented 20° in relation to the lateral margin of the jugal. The teeth are closely spaced, arranged in echelon. The two most anterior teeth are located medially, adjacent to the caniniform, and the remaining teeth follow posteriorly. The unworn elements have blade-like crowns, with strong ridges that join apically. A natural, crescentic facet is clearly distinct along the lingual margin of the new teeth. Worn teeth exhibit a widened, ellipsoid, occlusal surface, with two uneven wear platforms [8], fig. 2*b*,*c* (figure 6), a short and high labial facet and a lingual facet which is longer and lower than the former. The labial and lingual margins of each palatal tooth, below the wear facets, are nearly parallel. These teeth are implanted in distinct alveoli, indicating thecodonty. Two disarticulated molariform teeth were found in between the skull and the partially detached jaw, with their crowns posteroventrally oriented, and could represent upper teeth from the missing right side of the skull. They are shown as found in figures 4*a* and 5*a*. The pair (figure 6) is composed of an old tooth and a replacement tooth. The former is the most complete element of the two, measuring at least 11.5 mm in length. One of its borders, presumably the labial edge, is slightly concave, whereas its opposite margin is convex. It shows the characteristic crown morphology of the palatal teeth in *T. eccentricus*, with uneven wear facets. The taller wear facet is probably the labial one, assumed by comparison with the *in situ* left upper teeth. The crown is supported by a cervix that narrows towards the base, gradually becoming a long root. The end of the root is not preserved. The second tooth is less complete. It is a wide, leaf-shaped element that ends in a single cusp. Its apex is placed far below the top of the crown of the first tooth, suggesting that it was recently erupted. Both lingual and labial edges of this element are convex, but the presumably labial margin is slightly smoother. If the inferred orientation is correct, the existing cusp is on the labial margin. The apex shows no obvious traces of wear, and it was probably not yet in use at the moment of death. No lower teeth were recognized.

### 4.8 Axial elements

They are represented by two fragmentary ribs of parallel margins with no obvious curvature. The most complete fragment is 8 mm wide and 86 mm long.

### 4.9 Scapulocoracoid

**Figure 7.**
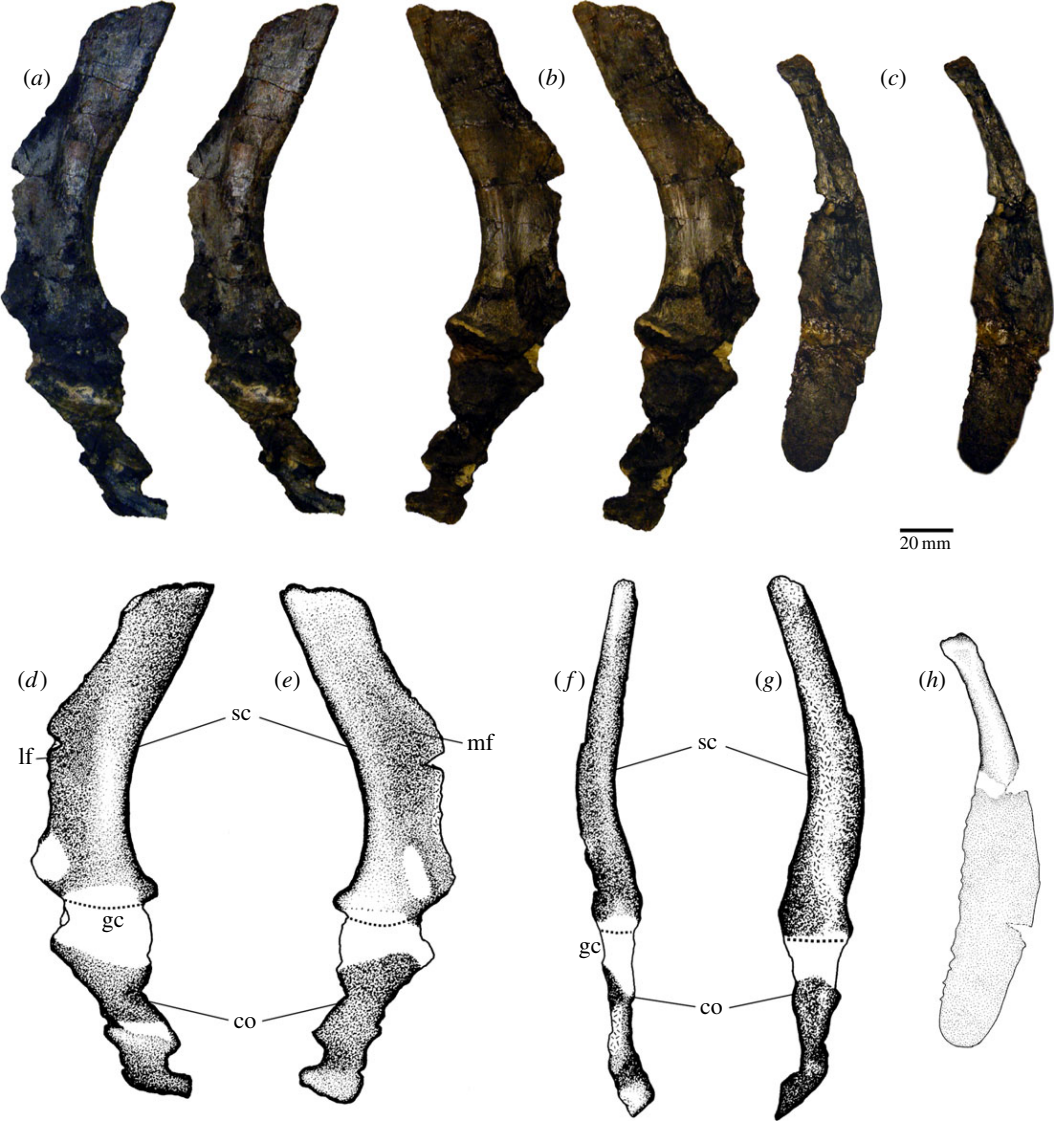
Left pectoral girdle of *T. eccentricus*. (*a*, *b*, *d*–*g*) Scapulocoracoid. (*a*) Lateral view, and (*b*) medial view, stereo-pairs. (*d*) Lateral view, (*e*) medial view, (*f*) posterior view and (*g*) anterior view, drawings. Clavicle in (*c*) medial view, stereo-pair, and (*h*) medial view, drawing. co, coracoid; gc, glenoid cavity; lf, lateral fossa; mf, medial fossa; sc, scapula.

The posterior portion of the scapular blade and part of the coracoid are present (figure 7). The posterior margin of the blade is concave posteriorly (figure 7*a*,*b*,*d*,*e*), and laterally presents a bulged area that extends beyond the middle height of the preserved blade (figure 7*a*,*g*). The remaining dorsal portion of the lateral face of the scapula is flat and smooth. There is a small portion of the blade preserved in front of the bulged area and clear evidence of the presence of a lateral fossa on the scapular blade, probably for the musculus deltoideus. The anterior portion of the scapular blade is not preserved. The scapula and the coracoid seem to contribute equally to the glenoid cavity, but this area is poorly preserved. Medially, the blade presents a shallow fossa in its basal portion and is flat dorsally (figure 7*b*,*e*). Part of the coracoid is articulated to the scapula, but its preservation is poor and very fragmentary.

### 4.10 Clavicle

It is a spatulate bone (figure 7*c*,*h*), showing an unusual extended, thin blade (approx. 1 mm) comprising around two-thirds of the preserved element. The remaining portion is a slightly curved, thick bar probably deformed due to compression. This portion may have been originally more curved. The distal portion of the bar is triangular in cross section. We estimate that the blade portion could represent half the total length of the bone.

### 4.11 Humerus

**Figure 8.**
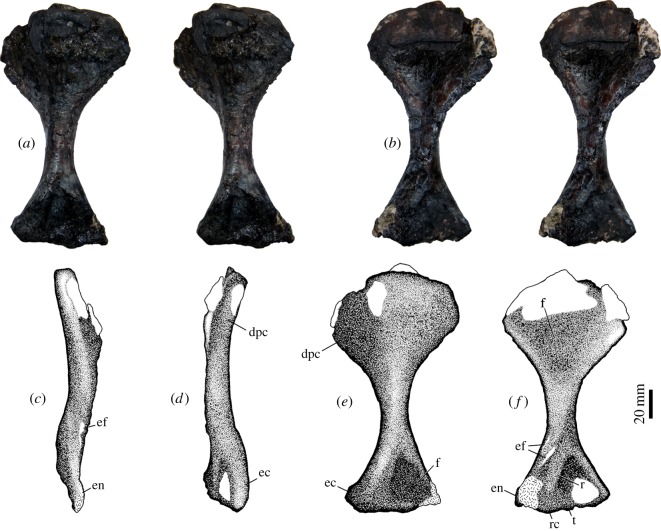
Left humerus of *T. eccentricus*. (*a*,*b*) Stereo-pairs in (*a*) dorsal and (*b*) ventral view. (*c*−*f*) Drawings in (*c*) medial, (*d*) lateral, (*e*) dorsal and (*f*) ventral views. dpc, deltopectoral crest; ec, ectepicondyle; ef, entepicondylar foramen; en, entepicondyle; f, fossa; r, ridge; rc, radial condyle; t, trochlea.

This is a moderately robust bone 177 mm in length (table 1), and with well-expanded proximal and distal portions, the former being conspicuously wider than the latter (figure 8). The bone underwent dorsoventral compression that mostly flattened the deltopectoral crest (figure 8*d*). The head is a thin structure (approx. 16 mm), slightly dorsally oriented. The angle of the preserved deltopectoral crest in relation with the axis running from the head to the medial portion of the bone, in proximal view, is approximately 35°. Taking into consideration the flattening of the bone, this angle would be somewhat higher in the undistorted bone. The deltopectoral crest is a nearly triangular plate extending half the length of the humerus (figure 8a,*b*,*e*,*f*). There is a shallow triangular fossa limited by the lateral margin of the deltopectoral crest and the medial margin of the proximal portion of the bone. There is a short shaft, ventrally flat and dorsally rounded, which extends from the level of the distal end of the deltopectoral crest to the entepicondylar foramen (figure 8a,*b*). The latter is elliptical, the medial opening having a length of 17 mm and the bar separating the medial of the lateral openings of the foramen presenting a maximum width of approx. 8 mm. The distal end of the bone is approximately an equilateral triangle (figure 8*a*,*b*,*e*,*f*). In dorsal view, the lateral margin of the distal portion presents an elongated bulge which becomes thicker distally (figure 8*e*). A slight elevation is observed in the medial edge of this portion of the bone and a shallow triangular fossa is placed between the lateral bulge and the medial elevation. In distal view, the ectepicondyle is more robust than the entepicondyle (although the latter is damaged). There is no ectepicondylar foramen. Ventrally there are clearly differentiated surfaces for radial and ulnar articulation. The partially exposed capitulum for the radius is globular and seems to have more articular area than the trochlea. However, the latter is encircling the distal edge of the bone and appears clearly exposed also dorsally. Ventrally, a thick ridge runs parallel to the medial margin of the distal end, producing a sulcus that ends in the entepicondylar foramen. No muscle scars could be identified.

**Table 1.** Postcranial measurements of *T. eccentricus*.

long bones	length (mm)	pes	length (mm)
humerus	178	digit i: metatarsal	16
humeral proximal end	98	digit i: first phalanx	11
humeral distal end	∼73	digit i: ungular phalanx	19
ulna	137	digit ii: metatarsal	24
ulna proximal end	42	digit ii: first phalanx	∼12
ulna distal end	34	digit ii: second phalanx	∼13
radius	128	digit ii: ungular phalanx	∼15
radius proximal end	27	digit iii: metatarsal	31
radius distal end	21	digit iii: first phalanx	16
tibia	∼159	digit iii: second phalanx	13
proximal end	∼39	digit iii: ungular phalanx	22
distal end	∼25	digit iv: metatarsal	∼38
clavicle	∼153	digit iv: distal tarsal	16
manus		digit iv: first phalanx	17
smaller digit: first phalanx	8	digit v: metatarsal	33
smaller digit: second phalanx	8	digit v: first phalanx	15
smaller digit: ungular phalanx	23		
larger digit: proximal phalanx	10		

### 4.12 Radius

**Figure 9.**
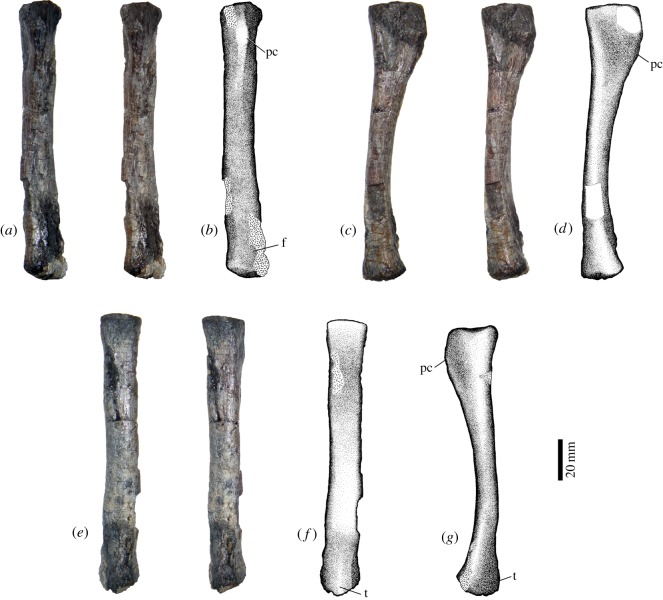
Left radius of *T*. *eccentricus*, stereo-pairs and drawings. (*a*,*b*) Posterior view, (*e*,*f*) anterior view, (*c*,*d*) lateral view and (*g*) medial view. pc, posterior crest; f, fossa; t, tuberosity.

It is 128 mm in length (table 1) with expanded, flat proximal and distal surfaces (figure 9). The proximal surface is clearly larger than the distal end (figure 9*c*,*d*). The proximal area for articulation with the capitulum is irregularly ovoid. There is a well-developed posterior crest (figure 9*a*,*b*) extending for more than a third of the bone length, lacking its proximal-most portion. Medial to this crest, there is a roughly triangular area for the contact with the ulnar notch. The shaft in the middle of the bone is elliptical, with its major axis (15 mm) being perpendicular to the longest axis of the proximal end. Distally, there is an elongated fossa (approx. 22 mm long) located in the posterior surface, close to the medial edge of the bone. On the lateral edge of the distal end, there is a moderate tuberosity for ulnar contact (figure 9*c*,*d*). The distal surface is subcircular (maximum length 21 mm).

### 4.13 Ulna

**Figure 10.**
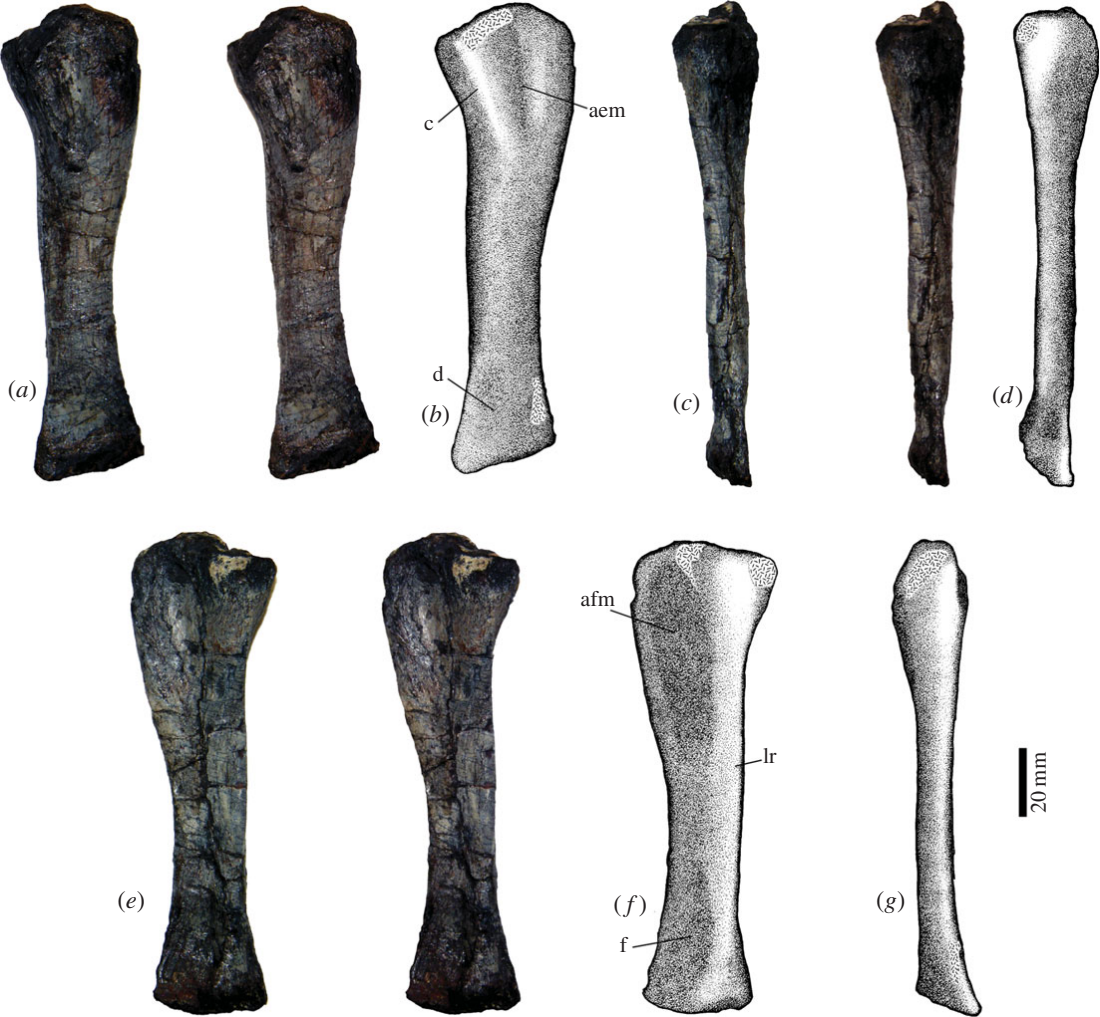
Left ulna of *T. eccentricus*, stereo-pairs and drawings. (*a*,*b*) Lateral view, (*c*,*d*) anterior view, (*e*,*f*) medial view and (*g*) posterior view. aem, attachment of extensor muscle; afm, attachment of flexor muscles; c, crest; d, depression; f, fossa; lr, longitudinal ridge.

This bone has been flattened (figure 10). It is slightly longer (137 mm) (table 1) and more robust than the radius. In lateral view, the proximal and distal ends are expanded, with the former being slightly larger than the latter. The bone shows a low torsion along its length, with the anterior margin of the bone of the proximal end located slightly medially and becoming lateral in the distal end. There is no ossified olecranon process, and the surface for articulation with the trochlea is oval and slightly concave. In the lateral surface, there is a well-defined crest, running distally from the trochlear facet for nearly a third of the bone length (figure 10*a*,*b*). The crest forms the posterior border of the radial notch and is delimited behind a shallow and elongated depression, probably related to the attachment of an extensor muscle [16]. This fossa has the same length as the crest. In the distal third of the lateral surface, there is a shallow, circular depression. On the medial surface, there is a prominent longitudinal ridge that starts in the trochlear facet and reaches the distal end of the bone (figure 10*e*,*f*). This elevation borders posteriorly a deep fossa extending for more than the third of the length of the bone, probably related with attachment of flexor muscles [16]. A second, distal fossa is also limited posteriorly by the ridge and extends through the distal third of the ulna. The distal end is remarkably compressed, being laminar, with a slight expansion near the anterior margin produced by the medial longitudinal ridge (figure 10*c*,*d*).

### 4.14 Manus

**Figure 11.**
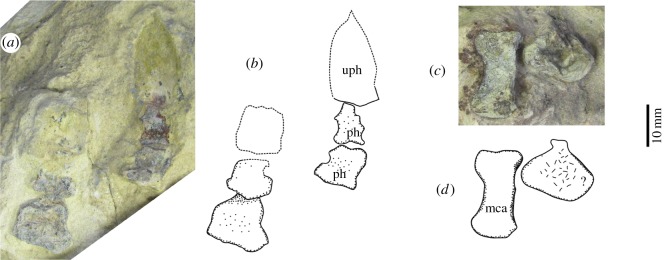
Left manus of *T*. *eccentricus*. (*a*) Photograph and (*b*) drawing of two digits. (*c*) Photograph and (*d*) drawing of a metacarpal and an unidentified bone found disarticulated. mca, metacarpal; ph, phalanx; uph, ungual phalanx; ?, unidentified bone.

There are at least two digits preserved (figure 11), one of them being smaller. These fragile elements have been uncovered from the matrix only on its dorsal surface. The smaller digit features three phalanges, including the ungual. The two non-terminal elements are robust and quadrangular, with a small constriction in the middle (8 mm long, 9 mm wide in the proximal phalanx; 8 mm long, 8 mm wide in the central element) (table 1). The dorsal surfaces of these phalanges are relatively flat. The ungual phalanx is partially preserved with its distal portion being represented as a natural mould. This phalanx is wider and much longer than the non-terminal elements (approx. 12 mm wide, 23 mm long), being ogival with a pointed end. The maximum width of this phalanx is proximal to its mid length.

The larger digit is represented by three phalanges. They may be non-terminals but their poor preservation precludes confident identification. The proximal element is rectangular and wider than it is long (16 mm wide, 10 mm long). The remaining elements appear to be sub-quadrangular.

Two additional bone elements (figure 11*c*,*d*) were found during preparation of the specimen, a few millimetres from the larger digit, below the distal ends of the ulna and the radius. Because they were not found in articulation, it is not clear if they are related to the larger digit or to another element. The larger bone is elongated (21.5 mm long, 11 mm wide) and is here interpreted as being a metacarpal. The distal end of this metacarpal is slightly concave and relatively expanded. The lateral margin of this metacarpal is very concave, in contrast to the medial margin, which, apart from the rounded medial projection of the distal end, appears straight. The proximal end of the metacarpal is slightly convex. The lateral margin of the proximal end is rounded and less broad than the lateral margin of the distal end. The convergence between the medial and the proximal margins of the proximal end appears as an oblique edge.

The second element is a wide trapezoidal bone (17 mm maximum dimension). The dorsal surface of the centre of the bone was damaged during detachment of the radius and the ulna. This bone features a small sub-quadrangular process, perpendicular to the axis of its maximum dimension. A similar projection is seen in both the ulnare and the radiale of *Galechirus scholtzi* [17], fig. 10. There are not enough elements to assess with confidence the identity of this bone. There is no evidence of disc-like phalanges being present in any of the preserved digits.

### 4.15 Tibia

**Figure 12.**
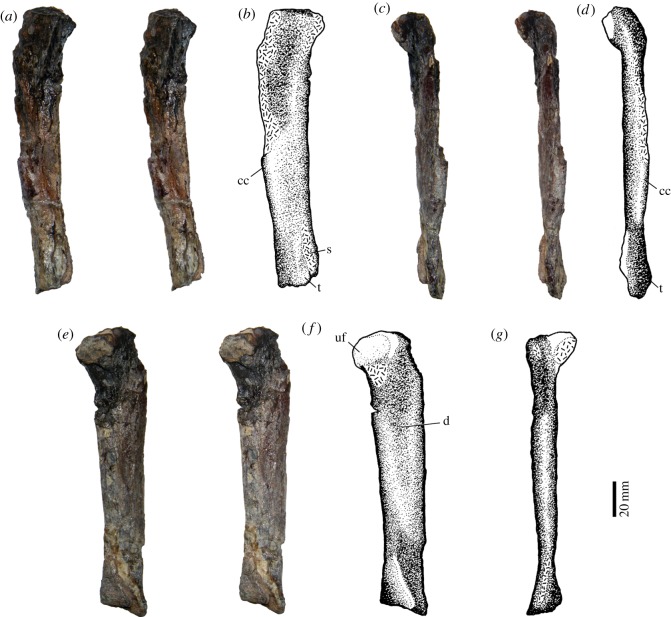
Left tibia of *T*. *eccentricus*, stereo-pairs and drawings. (*a*,*b*) Lateral view, (*c*,*d*) anterior view, (*e*,*f*) medial view and (*g*) posterior view. cc, cnemial crest; d, depression; s, sulcus; t, tuberosity; uf, unidentified bone fragment.

It is a long and slender bone (157 mm in length) (table 1), with the proximal half of its lateral surface eroded away (figure 12). The proximal end is expanded both anteroposteriorly and lateromedially. The maximum dimensions of these expansions are not known because of incompleteness of the bone. The cnemial crest is only partially preserved. The shaft is elliptical and mediolaterally compressed (25×12.5 mm). The only features observed in the distal half of the lateral surface are the presence of a prominent tuberosity, followed posteriorly by a strong longitudinal sulcus. Medially, the tibia shows a long, shallow depression, reaching the midpoint of the bone. The distal end is very flat and there is a triangular fragment of an unidentified bone firmly attached to it.

### 4.16 Pes

**Figure 13.**
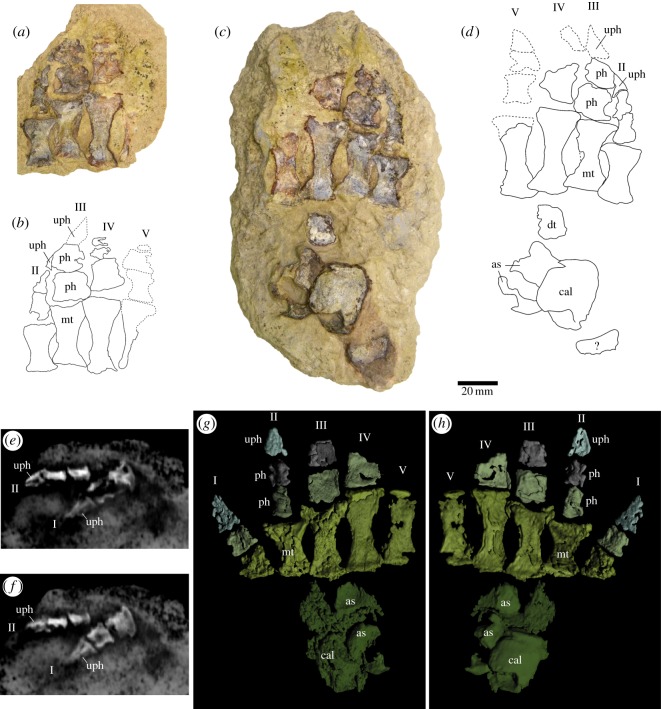
Left foot of *T. eccentricus*. (*a*–*d*) Rock slabs showing foot elements in basal cross section. (*a*,*b*) Palmar view, (*c*,*d*) dorsal view. (*e*,*f*) X-ray images showing digits 1 (dorsal view) and 2 (medial view). (*g*,*h*) Three-dimensional renderings of foot reconstructed from X-ray images: (*g*) palmar and (*h*) dorsal views. as, astragalus; cal, calcaneum; dt, distal tarsal; mt, metatarsal; ph, phalanx; uph, ungual phalanx; I–V, digit number.

The pes is represented by astragalus, calcaneum, fourth distal tarsal and five partial digits (figure 13*a*–*h*). The astragalus is a flat trapezoidal bone (figure 13*c*,*d*). The proximal edge of the bone, including the articulation facet for the tibia, is damaged. The calcaneum has the same anteroposterior length as the astragalus, but its lateral portion and most of the dorsal surface are missing. The fourth distal tarsal is basically a rectangle (16 mm long, 11 mm wide) (table 1), with the exposed surface partially eroded. The digits are contained in two counterparts of a block. Digits (iii)–(v) are exposed in coronal section (figure 13*a*,*c*), whereas digit (ii) lies turned around its longitudinal axis and its section is sagittal. The first digit is completely contained within the biggest counterslab, lying immediately ventral to the four remaining elements that were exposed through the opening of the rock, and it was only recognized after the CT scan was performed (figure 13*e*,*f*). All digits are robust. The first digit is represented by the metatarsal and two phalanges. The first metatarsal exhibits a different morphology from the remaining metatarsals, being quadrangular and robust (16 mm length). The next shortest metatarsal is the second (24 mm), the longest is the fourth (38 mm) and the third and fifth have similar lengths (31 mm and 33 mm, respectively). The distal portions of the metatarsals are mesolaterally more expanded than the proximal (figure 13*g*,*h*). The first phalanx from the first digit shows similar proportions to the corresponding metatarsal, being also wide and quadrangular (11 mm long and 10 mm wide). The first phalanx in the second digit, which is exposed in sagittal view, is relatively thin (12 mm long and 7 mm wide), and relatively narrow compared with the first phalanx of digits (iii) and (iv). The first phalanges from the third and fourth digits are quadrangular and larger (16 mm long and 14 mm wide; approx. 17 mm long and 16 mm wide, respectively) than in digit five, which is preserved as a natural cast (approx. 15 mm long and approx. 13 mm wide). The second phalanx in the second digit is approximately as long as the first one (approx. 13 mm long and 8 mm wide). The second phalanx of the third digit is roughly quadrangular (13 mm long×13 mm wide) with a strong constriction in the middle and a rectangular distal projection. The second phalanx in the fifth digit is a natural cast showing a faint outline (figure 13*a*–*d*). It appears to be narrower than the corresponding element in the third digit.

The ungual phalanx in the first digit is triangular (19 mm length and 12 mm proximal width) and elongated in dorsal view, showing a prominent dorsal ridge along its longitudinal axis. The ungual phalanges are preserved as a bone in digits one and two, and as natural moulds in the remaining elements. The phalanx of the second digit, in lateral view, is a bilaterally compressed claw (figure 13*g*,*h*), with a high, concave proximal articular facet, and a recurved, acute, distal end (approx. 15 mm in length and 10 mm in proximal width). The mould of the terminal phalanx in the third digit is triangular with an acute apex (approx. 22 mm long and approx. 12 mm wide). The moulds of the terminal phalanges in digits four and five are incomplete. There is no evidence of disc-like phalanges in any of the digits.

**Figure 14.**
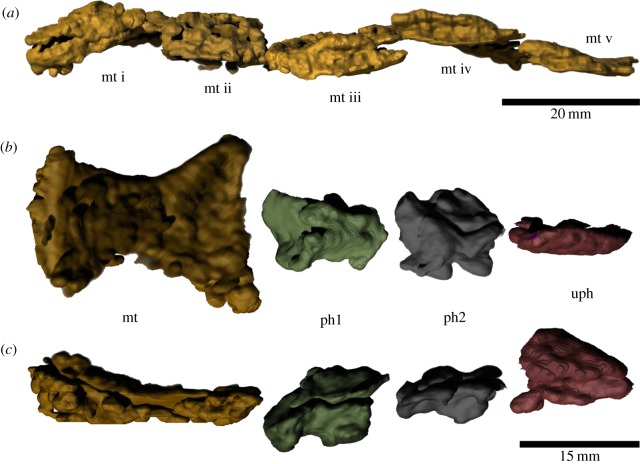
Left foot of *T. eccentricus*. (*a*) Proximal articular facets of the metatarsals. Metacarpal and phalanges of digit II in (*b*) dorsal and (*c*) lateral view. i–v metatarsal number; mt, metatarsal; ph1, first phalanx; ph2, second phalanx; uph, ungual phalanx.

An amphiarthrodial joint, more specifically synchondrosis, was probably present between metatarsal and tarsal bones. This is suggested by the flat proximal articulations of the metatarsals (figure 14). As this type of joint is separated by fibrocartilage, which limits movement between connected bones, we think that there was restricted movement between the metatarsal and tarsal bones.

Arthrodial joints are present between the distal metatarsal and proximal phalanges, as well as between the phalanges (figure 14). Given the concavity of the distal surface and the convexity of the proximal articular surface, we infer that this joint probably facilitated dorsoplantar movement. All of the metatarsals and phalanges are strongly dorsoventrally compressed, but this is most likely a taphonomical artefact.

### 4.17 Gastralia

**Figure 15.**
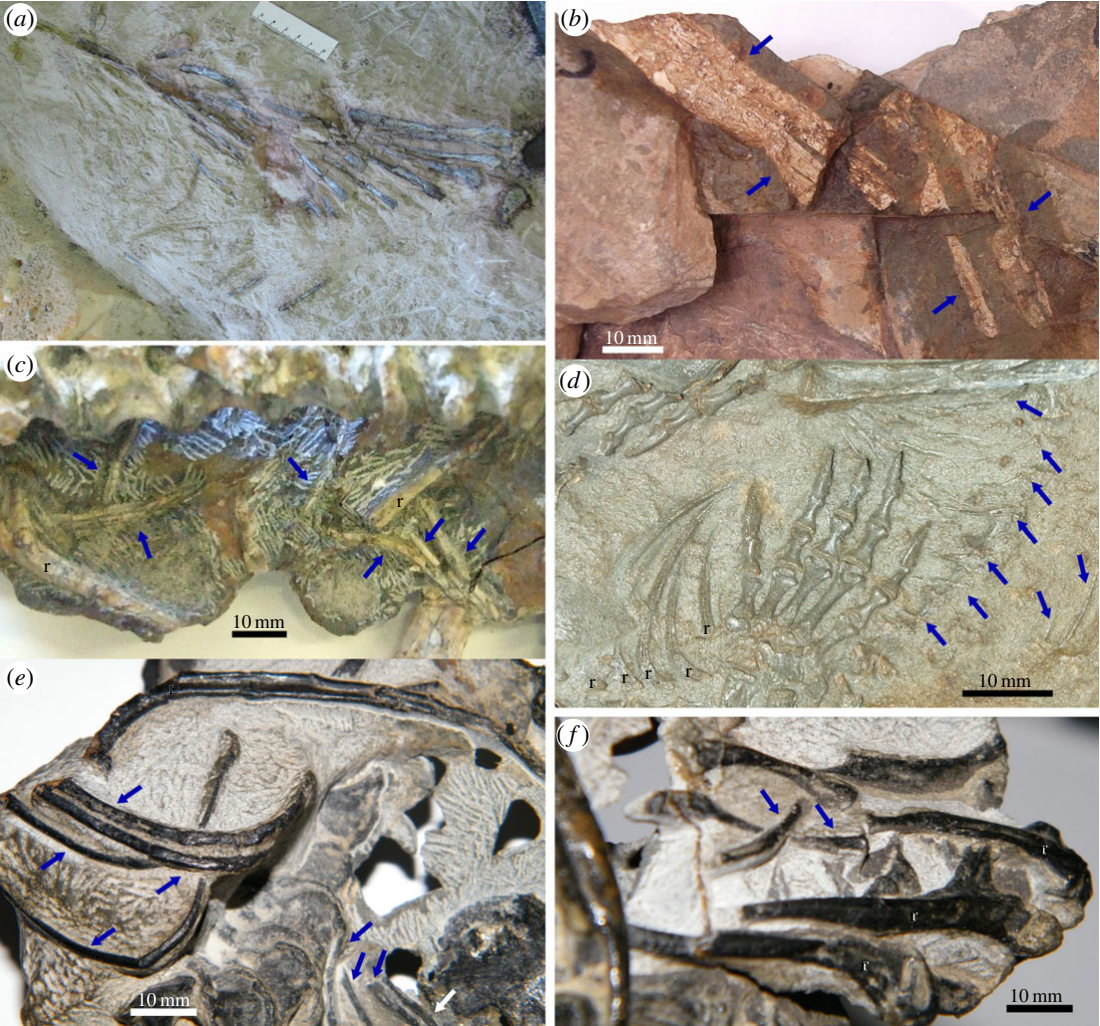
Gastralia of (*a*) *T*. *eccentricus*, (*b*) *A. africanus*, (*c*) gorgonopsian from the *Pristerognathus* Assemblage Zone (SAM-PK-K 10585), (*d*) basal anomodont *Galechirus*, (*e*,*f*) basal dicynodont *Eodicynodon* (NMQR 2991).

Dermal elements are represented by a set of at least 15 left and three right gastralia (figures 2*a*,*b* and 15*a*), preserved as long and very thin, delicate bones (generally less than 1 mm in thickness), and several more preserved as natural moulds. Each gastralium is mainly a straight rod with a slight proximal curvature. These bones show a somewhat expanded, spatulate proximal end varying between 6 and 15 mm in width. The distal portion width in the most complete gastralium is 3 mm. There is no contact between the proximal ends of these elements in the same side of the body, but they do converge distally to overlap each other. The complete preserved gastralia show that left and right elements appear to have a medial contact. They were probably recurved in life and appear now flattened due to lithostatic compression.

## 5. The dentition of *Anomocephalus africanus*

**Figure 16.**
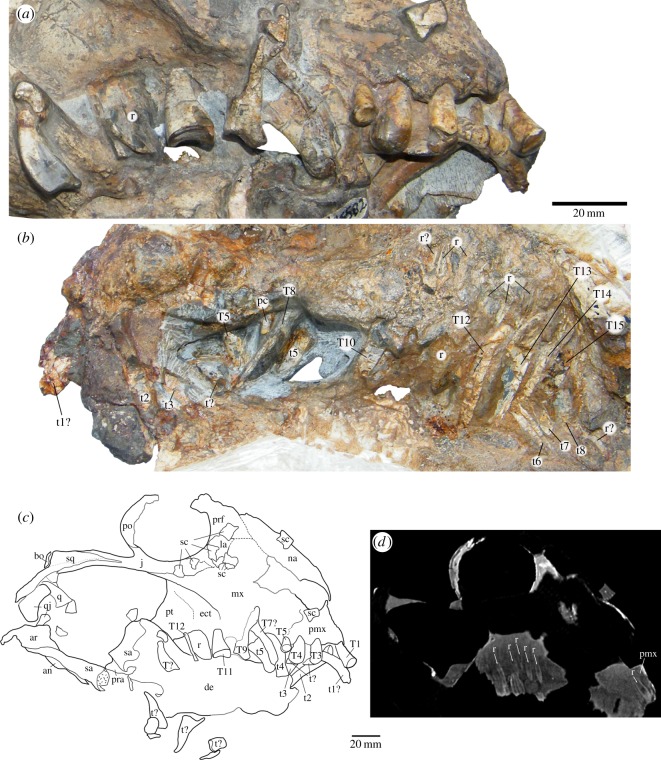
Dentition of *A. africanus.* (*a*) Lateral view; (*b*) medial view; (*c*) drawing of the cranium; (*d*) CT scan of the cranium showing replacement teeth (arrows). an, angular; ar, articular; bo, basioccipital; de, dentary; ect, ectopterygoid; j, jugal; la, lacrimal; mx, maxilla; n, nasal; pc, precaniniform; pmx, premaxilla; po, postorbital; pra, prearticular; prf, prefrontal; q, quadrate; qj, quadratojugal; r, replacement tooth; sa, surangular; sc, sclerotic ossicle; T, upper tooth; t, lower tooth.

The Karoo taxon possesses probably five upper incisors (figure 16*a*,*c*, T1–T5), the last one being the smallest and with a crown that seems ovoid-shaped in occlusal view. There is no evidence of a caniniform, and the dentition in the maxilla appears to begin with a tiny peg-like element (figure 16, pc) (possibly homologous to the small precaniniform found in *Tiarajudens*, although the tracing of the maxilla–premaxilla contact is tentative in both species), which is observable only on the medial view of the skull (figure 16*b*). This is followed by a buccolingually wide and mesiodistally short tooth (figure 16*b*, T8) after which there is a space without any teeth. At that level, there is a tooth out of place on the lateral surface of the maxilla (figure 16*a*,*c*, T9) and we interpret that the empty space was more likely the possible place of this tooth. Its crown exhibits uneven wear surfaces comparable with those seen in the palatal dentition of *Tiarajudens*. After the space in the medial view, it is possible to count at least six teeth (figure 16*b*, T10–T15, r) located on the pterygoid/epipterygoid, showing long and curved roots. Although the first tooth of this last series is in the alveolus, it is preserved outside of its natural position. The crown of this tooth is rectangular, with an occlusal basin that resembles that of traversodontid cynodonts, limited laterally by a high ridge (figure 16*a*,*c*). This is the tooth that better exposes the complete crown morphology of the palatal teeth. Considering the empty/damaged alveoli, it is possible to estimate that the complete right palatal dentition of *Anomocephalus* comprised at least 10 teeth.

The two most anterior *in situ* lower incisiforms (figure 16*c*, t2, t3) were previously noted and adequately described [18]. The first *in situ* dentary tooth is partially covered laterally by a displaced tooth (figure 16*c*, t1?) of similar morphology. As there is space in the anterior end of the jaw for at least one more tooth, this displaced element could represent the first lower incisiform. Another disarticulated tooth (figure 16*a*,*c*, t?), mostly visible in lateral view, lies horizontally, covering part of the cervix of the first *in situ* dentary tooth in lateral view, and lying above the crown of the second *in situ* dentary tooth. It is not clear if this is an upper or lower tooth. It seems to be relatively flattened, which suggests that it could be a transversally expanded tooth despite its crown not being exposed. After the two *in situ* lower incisiforms, there is a pair of slightly displaced lower teeth which are firmly attached to each other (figure 16, t4–t5). The morphology of the first tooth is not clear, but the second (t5) is a transversally expanded tooth rotated some 90° around its root–apical axis. This tooth is similar to its palatal counterparts, evincing a saddle-like crown. A long, vertical structure located between this pair of teeth is here interpreted as the weathered remains of a probable upper tooth. Three posterior lower teeth (figure 16*b*, t6–t18) are visible, in a bone that is tentatively identified as the dentary. An unerupted, replacement tooth is evident below the last lower tooth (figure 16*b*). These posterior lower teeth were preserved in tight occlusion against the last palatal molariforms. As far as it can be observed, the posterior lower teeth of *Anomocephalus* seem to have a similar morphology to their upper counterparts.

## 6. Discussion

### 6.1 Morphological comparisons

The skull dimensions of the Brazilian taxon are in agreement with *A. africanus* from the lower *Tapinocephalus* AZ of the South African Karoo. Both taxa are the largest basal anomodonts hitherto recorded, with cranial lengths larger than 210 mm. By comparison, *Biseridens qilianicus* from China is smaller, having a skull length estimated to be about 170 mm [19]. The Russian forms have variable skulls ranging between 54 and 150 mm long, whereas the remaining South African species are also small (table 2).

**Table 2.** Cranial length of basal anomodonts.

taxon	skull length (mm)
*Galepus jouberti*	>50
*Galechirus scholtzi*	>50
*Galeops whaitsi*	54
*Suminia getmanovi*	54
*Patranomodon nyaphulii*	∼55
*Otsheria netzvetajevi*	∼110
*Ulemica invisa*	∼150
*Biseridens qilianicus*	170
*Anomocephalus africanus*	210
*Tiarajudens eccentricus*	225

*Tiarajudens eccentricus* exhibits the typical skull morphology of basal anomodonts, i.e. short snout, large orbits and temporal fenestrae about the same size as or slightly larger than the orbit. Apart from similar skull lengths, other major similarities of the Brazilian taxon are shared with *A. africanus*. These include: (a) the jugal-squamosal arrangement in the zygomatic bar; (b) a triangular pterygoid bone in lateral view; (c) a very small, peg-like tooth in the anterior region of the maxilla; (d) an overall deep skull, due to ventral enlargement of tooth-bearing bones; (e) posterior placement of the quadrate in the squamosal bar; and (f) palatal teeth in the ectopterygoid and pterygoid, featuring thin necks and labiolingually expanded occlusal areas with deep, uneven basins. One of the autopomorphic characters of *Anomocephalus*, namely the tall, tongue-shaped coronoid eminence, might be present in the Brazilian taxon. Although the relevant area is not well preserved in *Tiarajudens*, an anterior dorsal expansion of the surangular may be interpreted as a coronoid eminence similar to the one observed in *Anomocephalus*. This leaves the presence of the oversized caniniform in *T. eccentricus* as the only trait that at present allows effective recognition between the two taxa. This character is interesting because it could also represent an expression of sexual dimorphism. However, we consider it premature to regard sexual variation as a valid hypothesis which explains the relationship between the two fossils, and favour treating these specimens, found at different basins, as two distinct taxa (see discussion below).

A unique feature of *Tiarajudens* in relation to all known therapsids, except dicynodonts, is the placement of the caniniform deep posteriorly in the snout, immediately in front of the orbit, being the last tooth in the margin of the maxilla. On the other hand, the considerably shorter snout and the large and widespread incisiform teeth of both anomocephaloids produce a peculiar condition in which nearly all the marginal dentition is composed of incisiforms.

### 6.2 Phylogenetic analysis

**Figure 17.**
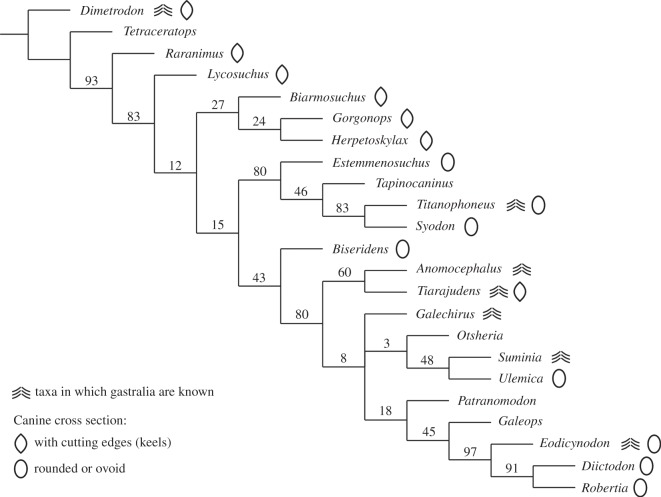
Cladistic analysis. Majority rule consensus from 15 most parsimonious trees (score 251) obtained on TNT [20]. Symmetric resampling support values (from 10 000 replicates, *p*=33) are provided before each node.

A cladistic analysis was run using a previously presented data matrix [8]. This dataset was augmented with five new characters: (81) gastralia (present and absent); (82) distribution of gastralia elements (packed and sparse); (83) enamel in caniniforms (present and absent); (84) caniniform cross section (slightly compressed lateromedially with a keel, rounded or ovoid, greatly compressed lateromedially, anteroposteriorly compressed); and (85) maxillae postcaniniforms (present and absent) (see the electronic supplementary material). Multistate characters were considered as non-additive (unordered). Based on additional preparation and CT scans, three character states were corrected for *Anomocephalus*: (52) dentary height in caniniform versus anterior postcaniniform regions (non-applicable [0→−]); (62) caniniform-like teeth in the upper dentition (absent [?→3]); and (63) canine-like teeth in the lower dentition (absent [−→2]). We decided to maintain the coding for character 69 (precaniniform maxillary teeth) as 0 (=present), despite the absence of caniniforms in *Anomocephalus*, because we interpret the tiny peg-like maxillary tooth of this taxon as homologous to the precaniniform found in *Tiarajudens*. We used the implicit searching strategy (i.e. shortest trees guaranteed) and obtained 15 most parsimonious trees with 251 steps. The majority rule consensus presented here (figure 17) shows the same topology previously reported in different studies [8,21–23]. The results confirm the sister-group relationship between *Tiarajudens* and *Anomocephalus*, and their basal position within Anomodontia, having *Biseridens* as out-group.

### 6.3 The record of gastralia in synapsids

Numerous instances of gastralia have been reported in different lineages of pelycosaur-grade synapsids. Thus, these elements are reported in caseasaurians, ophiacodontids, edaphosaurids and sphenacodontids as well as in varanopids [24–29]. By contrast, gastralia are rarely recorded in therapsids, being reported only in an anteosaurid dinocephalian, a gorgonopsian, and some basal anomodonts [18,30–32].

Most pelycosaur-grade synapsids show partial, displaced elements of the gastralia. The exception is represented in *Archaeothyris* and *Ophiacodon* in which the elements of the gastralia are preserved in their natural positions and arranged in a compact pattern, meaning that adjacent elements have a close contact anteroposteriorly [[24], text figs 4 and 5; [28,29]]. In addition, the rod-like elements on both sides contact proximally in *Archaeothyris*, whereas there is a midline chevron that connects with the lateral bones in *Ophiacodon* [24,33]. In these basal synapsid, each rod is formed by small scales which, in the case of *Archaeothyris*, are overlapped [24]. On the contrary, the arrangement of these elements in the South African varanopid *Heleosaurus* appears to be sparse, with spaces between successive rods. These spaces are twice the width of each rod. We assume this is the natural condition and not a taphonomic artefact, because the rods lie parallel to one another on both sides of the thorax [29], fig. 1 (photograph, specimen 1).

In therapsids, gastralia are known in the dinocephalian *Titanophoneus potens*, being described as thin rods, rounded in transversal section [30]. These elements are also known in the basal anomodonts *Galechirus* from South Africa [18] and *Suminia* from Russia [32]. In *Galechirus*, each gastralium is a slightly curved rod that ends in the midline. They appear to be widely separated from each other as there is only one rod per vertebra (figure 15*d*), although some rods are displaced. In a similar way, *Suminia* has a needle-like gastralium per trunk segment [32], resulting in wide spaces between the gastralia. Gastralia are also reported for the gorgonopsian *Viatkogorgon ivakhnenkoi* [31], where they are sparsely and similarly arranged as in the above-mentioned therapsids.

Besides these known cases, we found instances of gastralia in the most basal dicynodont *Eodicynodon* (NMQR 2991) and in a gorgonopsian from the *Pristerognathus* Assemblage Zone (SAM-PK-K 10585) (figure 15*c*,*e*,*f*). Each flat, thin and long gastralium in *Eodicynodon* overlaps a second element. We are uncertain if this contact is placed in the midline of the body, or if it is a division between medial and lateral scales as is known in *Ophiacodon* [34]. In the case of the South African gorgonopsian, the elements are thin rods located below the thoracic vertebrae (figure 15*c*). They are displaced and their arrangement cannot be assessed.

Some variation in the morphology of the gastralia can be highlighted. Only in some pelycosaur-grade synapsids are the rows forming the gastralia tightly assembled and lacking spaces between successive rods, thus forming a compact ventral shield. More sparsely arranged rods are observed in Middle and Late Permian synapsids, including the basal synapsid *Heleosaurus* and the therapsids *Galechirus* and *Suminia*. There are no records of therapsid gastralia with rods divided in smaller sections or ‘scales’. The presence of short scales, which is the primitive condition in tetrapods [35,36], is positively identified only in the Carboniferous *Archaeothyris* and in the Permo-Carboniferous *Ophiacodon* [34].

There is no evidence of the presence of gastralia in most therapsids. Being delicate skeletal elements and not articulated with the rest of the postcranium, they can easily be detached post-mortem and do not fossilize. Moreover, they are highly vulnerable to weathering and also to damage during the collection process, and due to their slenderness, they could be unnoticed during fossil collection. In addition, the collection of postcranial material in therapsids lags well behind the cranial material [37]. Thus, in several cases, the apparent absence of gastralia could be a result of taphonomical and collecting bias. Nevertheless, there are some small-to-medium size forms, with a high potential of preservation, and having several known postcrania (e.g. the cynodont *Thrinaxodon*, as well as several dicynodonts and therocephalians) in which it is clear that gastralia are absent. It is interesting to note that among therapsids, gastralia are most frequently preserved in basal anomodonts (figure 17). There are hitherto four taxa, including the two known anomocephaloids, in which these structures are known.

### 6.4 Dental adaptations for herbivory

Both anomocephaloids have a dentition that is indicative of a dedicated herbivorous diet. The leaf-shaped, coarse serrated incisors of *Tiarajudens* are readily comparable to those present in the Russian anomodont *Suminia*. This is a generic type of tooth that is present in a plethora of herbivore groups, having evolved several times among tetrapods. Teeth such as these are found in synapsids (several lineages), pareiasaur parareptiles, ornithischian and basal sauropodomorph dinosaurs, therizinosaurs, basal pseudosuchian archosaurs [38], notosuchian crocodyliforms [39] and extant iguanid lizards [5,6,40]. The function of these teeth was probably to pull and cut plant parts [40] in the same way extant iguanids do today. Caution has been raised [41] concerning an unequivocal interpretation of these kinds of teeth with strictly herbivorous habits. It has been remarked [41] that in several cases, these types of teeth are restricted to the posterior portion of the dental row, whereas anteriorly the teeth have a slight posterior curvature. The presence of these two different morphologies of teeth is more likely to represent an omnivorous type of diet [41]. The fact that the incisors are leaf-shaped and coarsely serrated, along with the morphology of the postcaniniforms (see below), is strongly suggestive of a herbivorous diet for *Tiarajudens*. In *Anomocephalus*, the premaxillary teeth are not leaf-shaped, instead having rounded crowns. This incisor morphology, however, is probably an artefact of tooth wear in the only known individual of this taxon.

The palatal teeth of both anomocephaloids are one of the most peculiar characteristics of this clade, as the presence of true molariforms in the palate was previously unknown in Synapsida. Their morphology is entirely consistent with a specialized, high-fibre herbivory. The broad wear surfaces observed in the palatal teeth of *Tiarajudens* are evidence that this taxon used them extensively for grinding fibrous food items. Moreover, these teeth are suggestive of a unique occlusion mechanism of palatal versus mandibular dentition, which is to a certain degree comparable to what is known in the basal synapsid *Edaphosaurus* [42]. The much greater number and overall simplified morphology of the teeth in the latter would preclude a precise occlusion like that allowed by the molariforms of the anomocephaloids. The lower dentition of *Tiarajudens* is not preserved. Fortunately, the corresponding lower jaw elements in *Anomocephalus* (figure 16*b*) are now visible in the medial aspect, being preserved in tight occlusion against the palatal molariforms. Thus, in the South African taxon, there is direct evidence of palatal-jaw tooth occlusion. This condition was almost certainly present in *Tiarajudens* as well, considering the strong similarity of tooth morphology in both taxa. In this way, all evidence supports the fact that anomocephaloids were dedicated herbivores, having developed an occlusion system that is unique within synapsids, and which provided the capability of oral processing of fibrous food items.

The morphology of the upper palatal teeth in *Anomocephalus* shows a flat basin and one of the margins of the tooth elevated. The teeth showing details of the crowns are out of place. We assume that in their natural positions, these teeth would have the elevated margin located labially, as in *Tiarajudens*. If that were the case, then the ecto-ental movement of the teeth during occlusion would be controlled by the lateral crest of the upper teeth, but there would be no impediment for an anteroposterior movement of the lower jaw during occlusion. The craniomandibular joint of *Anomocephalus* is partially preserved. There are two facets on the articular for the quadrate, aligned horizontally, the lateral one being slightly higher than the medial one, and set apart by a distinct step [18]. These facets are different from those of most anomodonts (and all dicynodonts) in that they are longitudinally extended and directed ventrally [43,44]. We propose that *Anomocephalus* (and perhaps anomocephaloids) had an incipient propaliny during the occlusion, as the longitudinal dimension of each facet for the quadrate is twice as great as its transversal dimension [18], and therefore would allow some fore and aft movement of the lower jaw. The mandibular movement in this taxon would certainly be more restricted than the extensive propaliny that is recognized in *Suminia* and dicynodonts [40,43,44], but comparable to the incipient propaliny proposed for *Ulemica* [43]. Propaliny is usually linked to improved capability for processing plant material [44,45].

### 6.5 Tooth replacement

There is evidence of palatal tooth replacement in both anomocephaloids. In *T. eccentricus*, substitution is evident in fourth, seventh, 10th and 13th teeth (figure 5*f*) and probably in an isolated tooth (figure 6). These teeth are recently erupted, arranged in a clear alternate fashion. In *A. africanus*, approximately seven replacement teeth are present (figure 16), representing at least two waves of substitution. Most of these elements had not yet erupted, but they became partially visible after removal of the outer surface of the palatal bones during opening of the plaster block that covered the medial view of the cranium. Others became clear in CT scan imaging (figure 16*d*). In addition, a palatal tooth that had fully erupted just prior to death is evident in lateral and medial views (figure 16*a*–*c*). This tooth possesses a leaf-shaped crown, being fully comparable with the replacement teeth observed in *Tiarajudens* (figures 5*f* and 6) and it represents an older wave of replacement in relation to the non-erupted teeth. There is also a replacement tooth in the premaxilla of the Karoo specimen (figure 16*d*), and probably another in the dentary, posteroventrally located in relation to the last lower tooth. The premaxillary replacement is located directly above an empty socket, after the first incisiform, indicating the substitution of an element that was discarded just prior to death. Overall, both anomocephaloids show a high rate of tooth replacement, which is consistent with the apparent heavy use of their teeth.

### 6.6 Function of the sabre-teeth

At first glance, the prominent caniniforms of *Tiarajudens* are probably the most visually remarkable characteristic of the taxon. The long and thin caniniforms of this anomocephaloid would appear to be fragile. However, the sabre-teeth of *Tiarajudens* are reniform in cross section along most of their length, structurally providing additional strength to the tooth. A thin layer of enamel renders additional protection to the caniniform, differing in this way from the tusks of dicynodonts [46–48]. In *Tiarajudens*, an extremely long root, reaching to almost the top of the skull (figure 5*b*), contrasts with the comparatively short root observed in dicynodont tusks [[46], fig. 16; [48], fig. 3] and ensures stability of the sabre-tooth during use.

Taking into consideration the herbivorous nature of the Anomocephaloidea, it is worthwhile considering the possible uses of these caniniforms as an aid to manage plant material. The absence of obvious signs of wear or natural damage on the preserved surface of the caniniform of *Tiarajudens* indicates that although this sabre-tooth could have been effectively employed to cut a number of food items without breaking, it was rarely employed in such a way, as no evident traces of use could be detected. This contrasts with the wear facets expected to be present if the caniniforms were used on a daily basis for digging, cutting or manipulating plant matter (e.g. stems, roots, leaves or bark). As mentioned above, the palatal teeth of both anomocephaloids were well adapted for processing high-fibre plant items that produced visible intensive wear. Thus, we conclude that the caniniforms of *T. eccentricus* were rarely, if ever, employed for feeding purposes.

**Figure 18.**
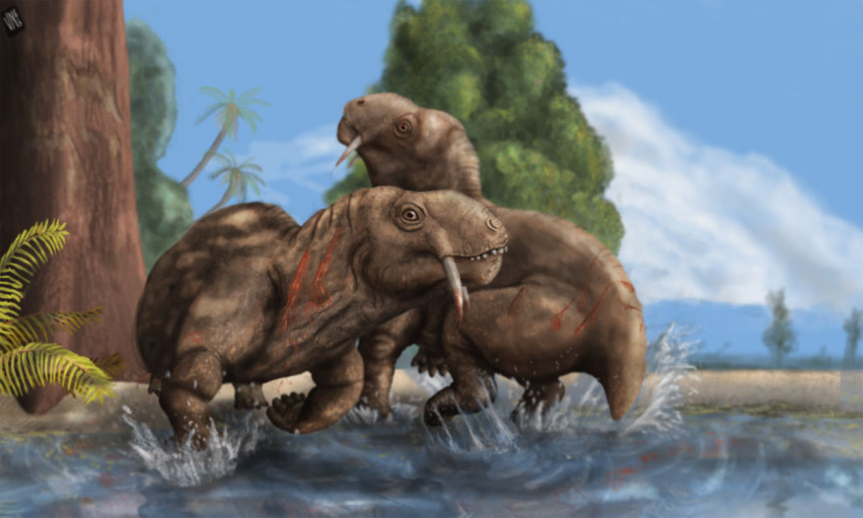
Artistic representation of two *T. eccentricus* individuals on agonistic behaviour in southern Brazil. Illustration by Voltaire Paes Neto.

A more likely interpretation for the presence of unusually large caniniforms in plant-eaters is that they were used for intraspecific display purposes. Modern herbivore analogues are found among deer [8], such as *Hydropotes* spp. (water-deer), *Moschus* spp. (musk-deer) and *Muntiacus* spp. (muntjacs). These mammals use their outsized caniniforms in male–male visual displays during agonistic encounters [49–52]. Occasionally, when the visual display fails to deter the opponent, males proceed to actually use their sabre-canines and try to cause harm to the opposition [49–53]. These deer use their canines to painfully scratch body surfaces, rather than inflicting deep wounds [50]. This strategy also minimizes the risk of losing their weapon during a fight, as would be the case if the canine perforated deeply the body of an opponent which then moved abruptly in order to escape [50]. Such kind of behaviour seems consistent with the morphological characteristics of the sabre-caniniforms of *Tiarajudens*. Intraspecific antagonistic encounters in modern tusked-deer are rare, and only evident in the mating season [51]. If this behaviour can be extrapolated to the Brazilian anomodont (figure 18), it would explain the absence of evident wear signs on the caniniforms.

The extremely large size of the caniniform in *Tiarajudens*, and the unique posterior insertion of its root, immediately anterior to the orbits, implies a huge effort required to open the lower jaw in order to present its caniniforms. This behaviour would not be necessary for visual display, as the enlarged caniniforms would be external to the entire lower jaw, including its soft tissues, being naturally exposed. Furthermore, this action would be unnecessary in order to perform caniniform blows to an opponent during physical combat [54].

The agonistic behaviour of visual display and/or physical combat with sabre-teeth observed in modern deer constitutes an alternative to head-butting and antler-sparring combat performed by ruminants possessing horns and antlers [50,52,55]. In this way, the proposed use of sabre-caniniforms in intraspecific display/fight by *Tiarajudens* constituted an alternative strategy to the head-butting combat employed by contemporaneous herbivorous dinocephalians [56]. If this inferred behaviour is correct, two classical alternatives of intraspecific combat among modern herbivores were already represented in plant-eater forms living in terrestrial ecosystems as early as the Middle Permian. It was at this time that the modern pattern of trophic interactions in terrestrial ecosystems, where a large and diverse population of herbivores supports a relatively small number of top carnivores, was established [5,6]. Hence, behavioural specializations that are considered so characteristic of Cenozoic mammals likely evolved at the time that the Earth's first complex herbivore terrestrial communities were constituted.

Considering that only males have tusks in modern and Tertiary deer, the possibility that the specimen addressed in this study may represent a male, needs consideration. Sexual dimorphism has been studied in some anomodonts: in *Aulacephalodon* males and females are known to differ in the shape and size of snout bosses [57], whereas in *Diictodon*, the presence of tusks has been interpreted as a male characteristic [48]. The conclusions drawn on these two Permian dicynodonts, however, were based on the morphometrical analyses of a large number of specimens. Although we consider that the enlarged caniniforms observed in *Tiarajudens* are a strong indication of sexual characteristics of a male individual, we recognize that this question cannot be positively solved while both anomocephaloids species are known only from very incomplete, holotypic material. The discovery of more specimens will hopefully help to shed light on this question and, at present, we prefer to recognize two taxonomic entities as a convenient, provisional hypothesis.

The sabre-caniniforms of *T. eccentricus* were also likely used in deterring attacks from predators [55]. In several mammals, morphological structures which function to deter predators are also used for intraspecific display or fight [55,58]. This is the case in the extant antlerless water-deer (*Hydropotes*) and musk-deer (*Moschus*) from Asia.

These unexpected features represented in *T. eccentricus* reinforce evidence of great morphological variation in basal anomodonts [59], despite their low taxonomic diversity, in contrast to the far more diverse dicynodonts which are remarkably conservative in their craniomandibular and, especially, dental morphology. The rise of anomodont morphological disparity occurred during the establishment of the modern trophic pyramid in the Guadalupian and was likely a key factor in the emergence of the stereotypic and successful dicynodont morphology, which allowed for the consolidation of this clade as the prominent herbivorous group and indeed the most species-rich vertebrate clade in Permian and Triassic times.

## 7. Conclusion

The Anomocephaloidea constitute a Gondwanan radiation of therapsids that independently acquired dental occlusion and developed new behavioural trends related to the acquisition of enlarged caniniforms. *Tiarajudens* and *Anomocephalus* represent highlights among the mosaic of evolutionary experimentations evinced by basal anomodonts. These experiments culminated with the explosion of dicynodonts, the most diverse tetrapod clade during the Permian and Triassic. Behavioural specializations, considered so characteristic of Cenozoic mammals, actually evolved at the time that the Earth's first complex herbivore terrestrial communities were established in the Guadalupian.

## Supplementary Material

Data matrix: Taxon/character-state matrix for phylogenetic analysis on TNT.

## Data accessibility

The datasets supporting this article have been uploaded as part of the electronic supplementary material.
